# Virtually Connected: Do Shared Novel Activities in Virtual Reality Enhance Self-Expansion and Relationship Quality?

**DOI:** 10.3390/bs15010067

**Published:** 2025-01-14

**Authors:** Rhonda N. Balzarini, Anya Sharma, Amy Muise

**Affiliations:** 1 Department of Psychology, Texas State University, San Marcos, TX 78666, USA; 2 Kinsey Institute, Indiana University, Bloomington, IN 47405, USA; 3 Department of Psychology, York University, Toronto, ON M3J 1P3, Canada; anyashar@yorku.ca (A.S.); muiseamy@yorku.ca (A.M.)

**Keywords:** virtual reality, self-expansion, romantic relationships, relationship satisfaction, intimacy, boredom

## Abstract

According to self-expansion theory, sharing novel experiences with a romantic partner can help prevent boredom and maintain relationship quality. However, in today’s globalized modern world, partners spend less time together and are more likely to live apart than in previous generations, limiting opportunities for shared novel experiences. In two in-lab experiments, we tested whether shared novel activities in virtual reality (VR) could facilitate self-expansion, reduce boredom, and enhance relationship quality. In Study 1, couples (*N* = 183) engaged in a shared novel and exciting activity in either VR or over video. Participants in the VR condition reported greater presence (i.e., felt like they were in the same space as their partner) and were less bored during the interaction compared to the video condition, though no main effects emerged for reports of self-expansion or relationship quality (relationship satisfaction and closeness). Consistent with predictions, people who reported more presence, in turn, reported greater self-expansion, less boredom, and greater relationship quality. In Study 2, couples (*N* = 141) engaged in a novel and exciting or a mundane experience in VR. Results were mixed such that participants in the novel VR condition reported less boredom and greater closeness post-interaction, though no effects emerged for self-expansion or relationship satisfaction. In exploratory analyses accounting for immersion, couples who engaged in the novel virtual experience reported more self-expansion, less boredom, and greater closeness. The findings suggest that virtual interactions may have less potential than in-person interaction to promote self-expansion but offer interesting future directions given VR’s ability to enhance presence beyond video interactions.

## 1. Introduction

The increasingly globalized modern world means that many people live apart from, or have less time with, those with whom they share the closest relationships (Amato & Hayes, 2013; Flood & Genadek, 2016; Holmes, 2010). In romantic relationships specifically, partners are more likely to live apart for job opportunities and educational pursuits than in previous generations (Hammonds et al., 2020; Holmes, 2010) and even among couples who live together, partners engage in fewer shared activities than they did 40 to 50 years ago (Amato et al., 2007; Dew, 2009; Kaufman-Parks et al., 2023). According to self-expansion theory, shared novel activities with a partner are important for warding off boredom and maintaining relationship quality (Aron & Aron, 1996, 1997; Aron et al., 2022). However, it is not yet clear if couples can engage in these types of experiences virtually. In the current research, we tested the potential of virtual reality (VR) to simulate novel shared experiences between romantic partners. In Study 1, we compared a novel experience in VR to the same experience over video chat to test whether the VR interaction increased presence (i.e., the feeling that they were in the same environment as their partner and experiencing the interaction together) and, in turn, greater self-expansion, lower boredom, and higher relationship quality. In Study 2, mirroring classic self-expansion experiments (Aron et al., 2022), we compared a novel experience in VR to a mundane experience in VR to test whether couples in the novel condition would report higher self-expansion, less boredom, and higher relationship quality. The findings have practical implications for couples seeking novel ways to connect and for researchers seeking ecologically valid ways to manipulate self-expansion.

### 1.1. Self-Expansion in Relationships

Spending quality time together is important for relationship maintenance (Jaremka et al., 2017), as shared positive leisure time helps couples maintain closeness and satisfaction in their relationships (Aron et al., 2000; Coulter & Malouff, 2013; Ogolsky et al., 2017; Rogge et al., 2013). However, certain activities may have greater potential to enhance relationship quality. Research on self-expansion has consistently shown that when couples engage in novel and exciting activities together (compared to activities that are more familiar or mundane), they experience less boredom and feel closer and more satisfied with their relationship (Aron et al., 2000, 2022).

In the seminal study on self-expansion, Aron and colleagues (Aron et al., 2000) assigned couples to either engage in a novel activity (e.g., an obstacle course) or a mundane activity together and found that couples who participated in novel and arousing activities reported less boredom and in turn, increased closeness and relationship satisfaction. Other experimental studies have shown similar effects; when couples are randomly assigned to engage in novel and exciting activities, they report boosts in their relationship quality compared to a waitlist control (Coulter & Malouff, 2013). In related homework-style studies, couples assigned to engage in novel and exciting (versus mundane but pleasant) activities report greater relationship quality; for a review, see Aron et al. (2022). For example, Reissman et al. (1993) assigned couples to participate in 1.5 h of exciting activities per week or 1.5 h per week of pleasant activities, or couples were assigned to a no-activity control condition for ten weeks. Participants assigned to the novel condition reported greater relationship quality than the other two conditions at the end of the study. Researchers have also used experience sampling designs to track partners’ novel shared activities in their daily lives. On days when couples engaged in activities that provided greater self-expansion—such as learning a new skill together (e.g., taking a cooking class), traveling together (e.g., going on a road trip), or engaging in adventurous sports (e.g., rock climbing)—both partners reported feeling more satisfied with their relationship on that day (Muise et al., 2019). In addition, couples who engaged in more self-expanding activities over the three-week study reported greater satisfaction three months later (Muise et al., 2019). Thus, strong support exists for the beneficial role of novel and exciting activities in promoting relationship quality in and outside a controlled laboratory environment.

Although self-expanding activities have been shown to help couples stay connected, maintain relationship satisfaction, and reduce boredom (Aron et al., 2022), previous research examining relational self-expansion has primarily investigated shared novel and exciting activities that involved partners interacting together, in person. Little is known about whether self-expansion can be facilitated virtually. Thus, one aim of the current research was to extend past work to test the potential of VR to facilitate self-expanding experiences for couples.

### 1.2. The Potential of Virtual Reality for Fostering Self-Expansion

Virtual reality (VR) is increasing in popularity and accessibility. Over 64 million Americans reported using VR in 2022 (Statista, 2024), and VR is projected to have 75.4 million American users by 2025 and 216 million users worldwide by 2025 (Digital in the Round, 2024; Lin, 2024; Sacks, 2024). In the US, the market is expected to keep expanding, especially as more immersive experiences and practical applications emerge (e.g., healthcare and training), helping VR reach a broader audience and drive long-term growth (Lin, 2024). Part of the success of VR has been its ability to immerse users into an experience. That is, VR technology is unique because it places users inside an experience, and users can immerse themselves in realistic simulations with virtual interaction partners. For example, a couple could visit Bali or take a virtual tour of the Taj Mahal together (Tracey, 2024), even from different geographical locations.

Given the immersive quality of VR (Howard et al., 2018; Valtchanov, 2010), researchers have been using VR to induce emotional experiences (Li et al., 2022; Meuleman & Rudrauf, 2018), such as anxiety and relaxation (Riva et al., 2007), fear and disgust (El Basbasse et al., 2023; Inozu et al., 2020; Ji et al., 2016), stress (Himes, 2024), and empathy (Marques et al., 2022; Tay et al., 2003), and simulate the presence of others (Froese et al., 2014; Sanchez-Vives & Slater, 2005; Slater et al., 2009) in a way that other text and video experiences cannot (Baym et al., 2004). Although using VR to facilitate connection in relationships is largely unexplored, in a survey of current VR users, the majority (77%) said they hope their future use of VR would involve more social interaction with other people (Koetsier, 2018). Nevertheless, there is little research on how VR interactions can enhance close relationships. Given the potential of VR to simulate the presence of a partner, there is also potential for relationship researchers to use VR to manipulate relationship-relevant experiences in more realistic ways. As such, understanding how shared experiences can be facilitated in VR has implications for relationship science and the maintenance of relationships more broadly.

### 1.3. The Current Study

In the current research, we draw on self-expansion theory (Aron & Aron, 1996, 1997) to examine whether VR can facilitate opportunities for couples to engage in novel shared activities. In Study 1, we compared a novel interaction between partners in VR to the same novel interaction over video to test whether VR experiences enhance a partner’s presence and, in turn, enhance self-expansion, reduce boredom, and promote higher relationship quality (i.e., closeness and relationship satisfaction). In Study 2, consistent with classic self-expansion experiments (Aron et al., 2000), we compared a novel experience in VR to a mundane experience in VR to test whether couples in the novel condition would report higher self-expansion and, in turn, less boredom and higher relationship quality. Given that shared novel experiences with a partner are a key predictor of closeness and satisfaction (Aron et al., 2022), the findings have practical implications for maintaining relationship quality over time and when partners are geographically distant. The hypotheses for Study 1 and Study 2 were pre-registered on the Open Science Framework (OSF).

## 2. Study 1

Study 1 aimed to compare people’s experience of a novel, shared activity with a romantic partner in VR to the same type of activity over video. Specifically, we tested whether couples who engage in a novel VR experience (compared to video) would report greater presence (H1)—feel like they were actually in the virtual environment with their partner—and higher self-expansion post-interaction (H2) and, in turn, report lower relational boredom and higher closeness and relationship satisfaction (H3). We also explored a series of auxiliary analyses to test whether the findings differed based on participants’ age, relationship length, or past VR experience.

### 2.1. Participants

Participants were recruited through the Undergraduate Research Participant Pool (URPP) at [BLIND FOR REVIEW] University and from the local community. At the time of our pre-registration, no studies had tested differences between shared novel experiences with a partner in VR compared to video interactions. Therefore, we obtained estimates of effect sizes from Campbell et al. (2020), a study of 100 participants that assessed team meetings in VR compared to video conferencing. The most relevant outcomes evaluated by Campbell et al. (2020) for the current study were presence (e.g., how much they felt like they were with the other users) and closeness (e.g., how close they felt to the other users). Presence (assessed on a 9-point scale) was higher in the VR condition (*M* men = 7.83, *M* women = 7.80) compared to the video condition (*M* men = 5.46, *M* women = 5.84; *F* = 58.40, *d* = 1.5284). Closeness (assessed on a 9-point scale) was also higher in the VR condition (*M* men = 6.61, *M* women = 6.57) compared to the video condition (*M* men = 4.66, *M* women = 4.80; *F* = 26.93, *d* = 1.0379). Using G*Power 3.1 (Faul et al., 2009) for a two-tailed test for an effect size of 1.0379 (the most conservation effect) with 99% power and 0.05 alpha, 70 couples (35 per condition) would be needed. However, we oversampled to account for attrition, failed attention checks, or potential technical issues. We initially recruited 111 couples (*N* = 222). In line with our pre-registration, we removed 18 couples (*N* = 36) who experienced technical issues during the study, did not follow instructions (i.e., removed headsets, left the virtual area), or failed the attention checks (e.g., a question asking participants to select the type of virtual activity they engaged in).

Our final sample included 186 participants (*N* = 93 couples), including 92 men, 93 women, and one non-binary participant. The final sample was racially diverse, though most participants identified as white (37.1%) or black (16.7%). The majority of participants identified as straight/heterosexual (74.7%) and reported “seriously dating” their partners (88.2%). Participants were in established relationships with their partners for approximately two years (*M* = 2.22 years, *SD* = 2.50) and were in their early 20s (*M* = 20.2, *SD* = 3.81) on average. See Table 1 for sample demographics.

### 2.2. Procedures

Before enrollment in the study, informed consent was obtained from all participants. Once both partners agreed to participate, they were asked to complete a 20 min online survey before attending the in-lab session, which included demographic and general relationship questions, and questions about previous experiences with VR. For the in-lab session, couples were randomly assigned to one of two conditions: (1) the novel VR experience condition (*N* = 82; 41 couples) in which participants took a virtual hot air balloon ride over a Kenyan safari, or (2) the novel video experience condition (*N* = 104; 52 couples), in which participants watched a video of a hot air balloon ride over a Kenyan safari (via a video chat with their partner). In both conditions, partners were taken to separate rooms and interacted virtually using VR or video chat. The interactions lasted approximately six minutes in both conditions. Following the interaction, participants completed a post-manipulation survey asking about their feelings during the interaction. Partners were then reunited and debriefed.

### 2.3. Measures

**Figure 1:**
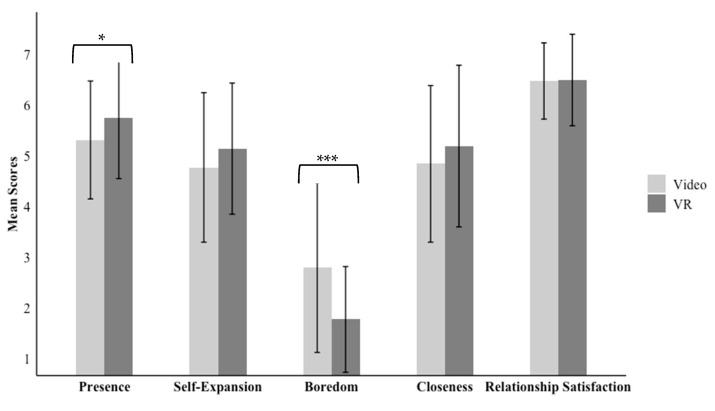
Descriptive statistics between conditions for Study 1 focal variables. Note: The error bars represent standard deviations, and significance is denoted with *** *p* < 0.001 and * *p* < 0.05.

After exposure to the experimental condition, participants were asked to respond to a series of post-manipulation questions outlined below. Table 2 shows correlations between variables within each condition, and Figure 1 reports means across conditions.

#### 2.3.1. Presence

Participants reported their sense of their partner’s presence during the interaction, e.g., “How much did you have the sense that you were in the environment with your partner?”; “How much did you feel like you and your partner were having a shared experience (i.e., experiencing the same thing at the same time)?”, and “During the interaction, did you feel you were with your partner (like your partner was present)?”, adapted from the Networked Minds Social Presence Inventory (NMSPI) (Biocca & Harms, 2024). Possible responses were on a 7-point scale (Q1, 1 = *not at all*, 7 = *completely*; Q2–3, 1 = *never*, 7 = *all the time*), and the items were mean aggregated, with higher scores indicating higher perceived presence.

#### 2.3.2. Self-Expansion

Participants were asked to rate their perceptions of self-expansion with their partner during the interaction. Participants were provided the following instructions: “Thinking about the experience you and your partner just had together, answer each question according to the way you personally feel, using the following scale”. Participants were then asked to rate six items that were adapted from the Self-Expansion Scale (Lewandowski & Aron, 2002) (e.g., “How much did your interaction with your partner result in your having new experiences?”; “How much has the experience with your partner today resulted in your learning new things?”). Responses were provided on a 7-point scale (1 = *not very much*, 7 = *very much*), and the items were mean aggregated, with higher scores indicating higher self-expansion post-interaction (α VR = 0.90; α video = 0.90).

#### 2.3.3. Boredom

Post-interaction, participants were asked to indicate how bored they felt during the interaction (e.g., “To what extent did you feel bored during the interaction?”). Responses were provided on a 7-point scale (1 = *not very much*, 7 = *a great deal*) with higher scores indicating higher boredom.

#### 2.3.4. Closeness

The Inclusion of Other in the Self Scale (IOS) (Aron et al., 1992) was used to assess perceptions of closeness and intimacy with their partner during the interaction. Participants were asked to indicate how close they were with their partner (e.g., “How interconnected were you and your partner during your interaction?”) using a widely used single-item pictorial scale that provides partially overlapping circles to indicate closeness with the circles increasing in closeness as they become more inclusive of the other in their self. Participant’s selections were assigned a value that ranged from 1 (indicating the “*most distant*” via the overlapping circles) to 7 (indicating the “*most close*” via the overlapping circles). This scale has shown test–retest reliability as well as convergent, divergent, and criterion validity (Aron et al., 1992).

#### 2.3.5. Relationship Satisfaction

Post-interaction, participants were asked to indicate their satisfaction with their romantic partner. One item assessed relationship satisfaction (e.g., “How satisfied were you with your relationship?”). Possible responses were on a 7-point scale (1 = *not at all*, 7 = *extremely*), with higher scores indicating more satisfaction.

#### 2.3.6. Experience with VR

Participants were asked to indicate their previous VR experience on a 4-point scale (e.g., “Besides today, how much experience have you had with virtual reality?”; 0 = *none*, 1 = *used it once*, 2 = *used it a few times*, 3 = *I own a VR/use VR frequently*) post-interaction, with higher scores indicating more experience using VR. Most participants had some experience with VR, with only 19.4% of participants indicating no prior experience, 27.4% indicated using it once before, 39.2% indicating that they had used it a few times, and 8.1% indicated that they either owned a VR headset or used VR frequently (with 5.9% not answering the question).

### 2.4. Analytic Approach

To examine whether people in the novel VR condition (compared to those in the novel video condition) reported greater presence (H1) and self-expansion (H2), and to exploratorily examine the effects on boredom, closeness, and relationship satisfaction (E1–3), we conducted a series of multilevel models in which partners were nested within couples.^1^ We used multilevel modeling (mlm) to test the effect of the condition on the outcomes of interest to account for the fact that our data include both partners in a relationship having a shared interaction. In these analyses, we tested whether the experimental condition (0 = *video*, 1 = *VR*, a dyadic variable given that partners are assigned to the same condition) predicted our outcomes of interest (examining each outcome individually). Then, we tested whether couples in the novel VR condition reported greater self-expansion, less boredom, and greater relationship quality post-interaction through presence (H3). To do so, we conducted a series of multilevel mediation models using the Monte Carlo Method for Assessing Mediation (MCMAM) (Selig & Preacher, 2024). Indirect effects were deemed significant if the 95% CI did not contain zero. In auxiliary analyses, we examined whether the effect of the condition on our key outcomes was moderated by relationship length, age, or previous experience with VR. This involved adding an interaction between the condition and each moderator (examined individually). If the interaction was significant, we interpreted the effects at high and low levels of the moderator. The data and syntax for all analyses reported for this paper can be found on the OSF.

### 2.5. Results

#### 2.5.1. Shared Novel Experiences in VR Versus Video

Consistent with our pre-registered predictions, people who engaged in a brief novel interaction with their partner in VR (*M* = 5.74, *SD* = 1.19) reported a greater sense of presence than people in the video condition (*M* = 5.31, *SD* = 1.16; *b* = 0.41, *t*(84.42) = 2.01, *p* = 0.048, 95% CI [0.004, 0.81]). However, inconsistent with our predictions, although people in the VR condition reported higher self-expansion (*M* = 5.14, *SD* = 1.30) compared to those in the video condition (*M* = 4.77, *SD* = 1.47), the difference between the conditions was not significant (*b* = 0.37, *t*(88.68) = 1.60, *p* = 0.113, 95% CI [−0.09, 0.83]).

Next, in exploratory analyses, we examined the effect of the condition on boredom, closeness, and relationship satisfaction post-interaction. We found significant differences in condition for people’s reports of relational boredom (*b* = −1.03, *t*(91.14) = −4.72, *p* < 0.001, 95% CI [−1.46, −0.60]), such that people in the VR condition reported lower boredom (*M* = 1.78, *SD* = 1.04) than people in the video condition (*M* = 2.80, *SD* = 1.68) post-interaction, but no significant differences emerged between the conditions and people’s reports of closeness (*p* = 0.213) or relationship satisfaction (*p* = 0.909) post-interaction.

#### 2.5.2. Mediation Through Presence

**Figure 2:**
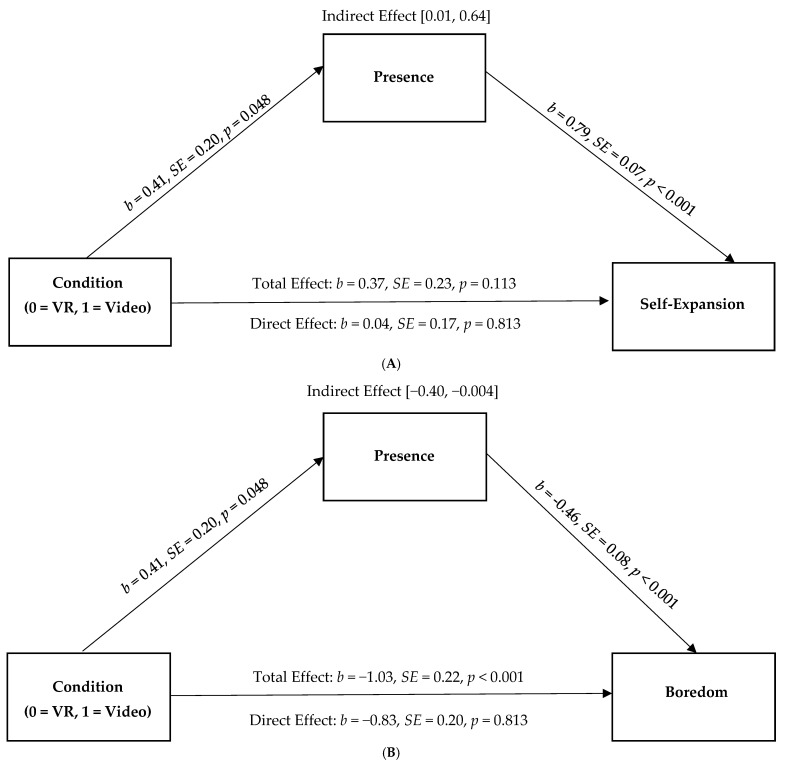

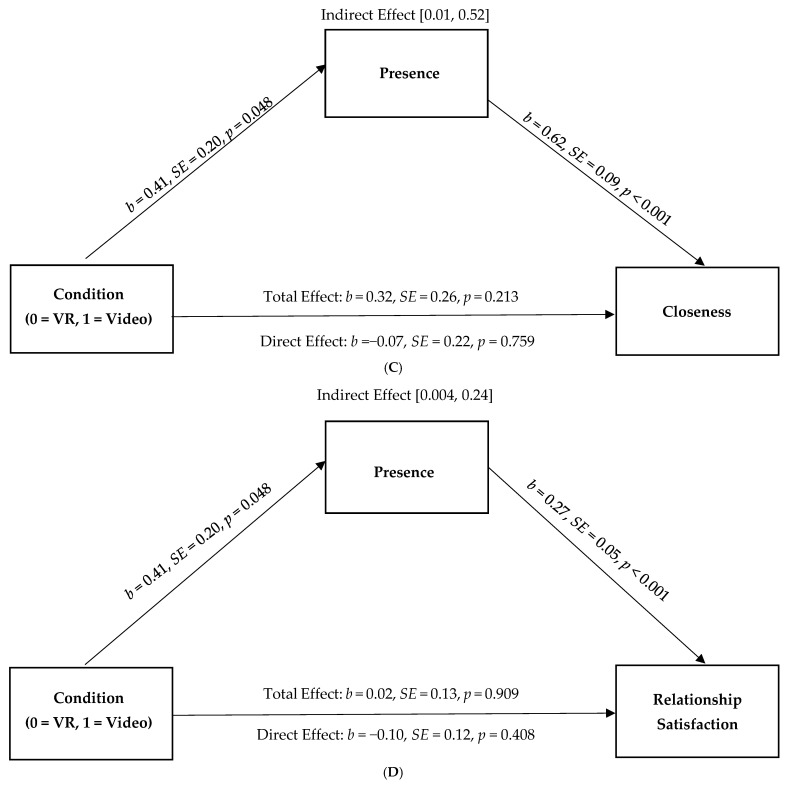
Perceived partner presence as a mediator of the association between condition (0 = VR, 1 = video) on reports of (**A**) self-expansion, (**B**) boredom, (**C**), closeness, and (**D**) relationship satisfaction in Study 1.

Next, consistent with our pre-registration, we tested whether people benefit from novel virtual experiences with their partner (compared to novel experiences over video chat) because their partner was perceived to be more present in VR (i.e., they felt more like they were with a partner). We examined whether the greater presence reported by participants in the novel VR condition would, in turn, be associated with higher self-expansion, less boredom, higher relationship satisfaction, and greater closeness. Consistent with predictions, people in the VR condition reported more presence than those in the video condition, which was, in turn, lower boredom (95% CI [−0.40, −0.004]). Although there were no direct effects on self-expansion, relationship satisfaction, and closeness, there were indirect effects via greater presence on higher self-expansion (95% CI [0.01, 0.64]), greater closeness (95% CI [0.01, 0.52]), and higher relationship satisfaction (95% CI [0.004, 0.24]) post-interaction. (See Figure 2A–D).

#### 2.5.3. Considering the Role of Relationship Length, Age, and Experience with VR

In auxiliary analyses, we explored the role of relationship length, age, and experience with VR in the effect of the condition on our key outcomes. We examined each as a separate moderator. No significant differences by relationship length emerged (all *p* > 0.05). However, we found that the association between condition and closeness was moderated by age (*b* = 0.23, *t*(114.92) = 3.06, *p* = 0.003, 95% CI [0.08, 0.38]), such that older participants (+1 SD) reported greater closeness in the VR condition compared to the video condition (*b* = 1.05, *t*(97.09) = 2.63, *p* = 0.010, 95% CI [0.26, 1.84]), whereas there was no effect of the condition for younger participants (−1 SD; *b* = −0.71, *t*(100.75) = −1.81, *p* = 0.073, 95% CI [−1.50, 0.07]). Participants’ age did not moderate the association between the condition and other focal variables. The effect of the condition on self-expansion was moderated by having prior experience with VR (*b* = −0.53, *t*(170.11) = −2.25, *p* = 0.026, 95% CI [−0.99, −0.06]), such that people with less experience with VR (−1 SD) reported greater self-expansion in the VR condition compared to the video condition (*b* = 0.74, *t*(122.69) = 2.31, *p* = 0.022, 95% CI [0.11, 1.37]), whereas there was no effect of the condition on reports of self-expansion among people who had more experience with VR (+1 SD; *b* = −0.22, *t*(124.13) = −0.68, *p* = 0.499, 95% CI [−0.85, 0.42]). We also found that the effect of the condition and closeness was moderated by past VR experience (*b* = −0.57, *t*(168.91) = −2.18, *p* = 0.031, 95% CI [−1.09, −0.05]), such that people with less experience with VR (−1SD) reported greater closeness in the VR condition compared to the video condition (*b* = 0.73, *t*(121.05) = 2.06, *p* = 0.031, 95% CI [0.03, 1.43]), but, there was no effect of the condition on people who had more (+ 1SD) experience with VR (*b* = −0.31, *t*(122.51) = −0.87, *p* = 0.388, 95% CI [−1.01, 0.40]).

### 2.6. Study 1 Discussion

The results from Study 1 suggest that people who engaged in a brief novel interaction with their partner in VR experienced a greater sense of presence than people who had a novel interaction over video. However, inconsistent with our predictions, people in the VR condition did not report greater self-expansion post-interaction. It is possible that participants’ experiences in the novel VR and video condition did not result in differences in self-expansion because both experiences were “novel”. However, participants reported less boredom during the VR experience compared to the video interaction with their partner. Greater presence in the VR (versus video) condition accounted for the lower boredom reported in the VR condition. There were significant indirect effects via presence on self-expansion, relationship satisfaction, and closeness, but these emerged without significant direct effects. Importantly, however, in exploratory analyses testing the role of relationship length, age, and VR experience, people in the VR condition did report greater self-expansion and closeness with their partner, but only when they had less experience with VR; there were no effects for those with more experience. Also, participants who were older reported greater closeness following the interaction in the VR versus video condition, but there was no effect for younger participants. Therefore, the VR condition may have been more novel for people with limited previous experience with VR or for those who were older, leading to stronger effects on self-expansion and closeness.

## 3. Study 2

Study 1 demonstrated that a VR interaction could enhance the presence of a partner (feeling like you are having a shared experience with a partner) compared to a video interaction and that perceiving a partner to be more present, in turn, was associated with higher self-expansion, less boredom, and higher relationship quality post-interaction. However, when we examined the main effects in the overall sample, people in the novel virtual condition did not differ in their reports of self-expansion from participants in the video condition. It is possible we did not find the expected main effect of the condition on self-expansion overall because both conditions involved a novel experience with a partner, or differences may exist between conditions but not captured with our current sample size (we based the sample size on a study examining VR versus video interactions among work colleagues and the effects on closeness and related outcomes might be smaller for romantic partners). As such, in our next study, we aimed to manipulate the novelty of a shared interaction rather than the communication technology and to recruit a larger sample. In Study 2, all participants interacted using VR, and we manipulated the type of interaction (novel versus mundane) to mirror previous in-lab studies of self-expansion (Aron et al., 2000). We aimed to test whether engaging in a novel and exciting activity compared to a mundane but pleasant activity with a romantic partner (both of which occurred in VR) would enhance self-expansion and, in turn, reduce boredom and enhance relationship quality. More specifically, we predicted that couples in the novel condition would report greater self-expansion (H1), less boredom, greater closeness, and greater relationship satisfaction (H2) than people in the mundane VR condition. We also predicted that higher self-expansion in the novelty versus the mundane VR condition would mediate (account for) the effect of the condition on lower boredom, greater closeness, and greater relationship satisfaction.

Results from Study 1 suggest that VR enhances feelings of presence; however, in Study 2, both conditions were in VR, and thus, we expected presence to be high and not differ across conditions. As such, in this study, we were interested in whether differences emerged among those who had a novel versus mundane experience with their romantic partner in VR. We pre-registered our predictions before data collection (see the OSF); however, we also tested a series of exploratory analyses that deviated from the pre-registered hypotheses. To start, we assessed immersion, the extent to which people feel engaged in the virtual environment and can block out the physical world, which is important for people’s experiences in VR (Berkman & Akan, 2019; Cummings & Bailenson, 2016; Maymon et al., 2023). In these auxiliary analyses, we sought to account for immersion (Berkman & Akan, 2019) and examine whether the effects of interest emerged when the participant’s engagement with the task was held constant. Lastly, as in Study 1, we also explored a series of auxiliary analyses to test whether the findings differed based on participants’ age, relationship length, or past VR experience.

### 3.1. Participants

Participants were recruited through the Undergraduate Research Participant Pool (URPP) at York University. At the time of our pre-registration, no studies had tested differences between self-expansion in relationships in VR. However, a previous in-lab study compared couples’ reports of relationship satisfaction after engaging in a novel and exciting versus a mundane activity in person. This study reported large effect sizes (*d* = 0.93) on relationship satisfaction (Aron et al., 2000). When we estimated an effect of *d* = 0.93 with 80% power and an alpha of 0.05 using G*Power 3.1 (Faul et al., 2009), it was estimated that we would need 40 couples (20 per condition). However, because this effect size is derived from a study in the lab (and not in VR), we wanted to estimate a more conservative, medium effect size. With a medium effect (0.5) with 80% power and 0.05 alpha, 128 couples (64 per condition) would be needed. We oversampled by approximately 10% to account for attrition, failed attention checks, or potential technical issues.

We initially recruited 159 couples (*N* = 318); however, we removed 14 couples (*N* = 28) who experienced technical issues during the study or did not follow instructions (i.e., removed headset, left virtual area). In line with our pre-registration, we removed three participants for failing the attention check to ask them to select the type of virtual activity they engaged in and one participant whose post-manipulation data were not saved. In these four cases, their partner’s data was retained. Our final sample included 287 participants (*N* = 142 couples, three individuals), 128 men, 156 women, and three non-binary people. The final sample was racially diverse, though most participants identified as South Asian (25.78%) or white (24.39%). Most of the participants identified as straight/heterosexual (75.26%) and reported “seriously dating” their partners (79.09%). Participants were in established relationships with their partners of nearly two years (*M* = 1.83 years; *SD* = 1.77 years) and in their early 20s (ranging from 17 to 41, *M* = 20.3, *SD* = 3.64) on average. See Table 1 for demographic information.

### 3.2. Procedure

Once both partners agreed to participate in the study, they were asked to complete a 20 min online survey before attending the in-lab session. The online survey included demographic and general relationship questions, and questions about previous experiences with VR. Couples were then asked to attend an in-lab session. Once in the lab, partners were taken to separate rooms and were randomly assigned to have one of two types of interactions using VR: (1) a shared novel experience (a gondola ride in the Swiss Alps) (*N* = 148; 73 couples, two individuals) or (2) a shared mundane experience (a virtual porch which overlooked a scenic environment) (*N* = 138; 68 couples, two individuals). The interactions lasted approximately 6 min in both conditions and following the interaction, participants completed a post-manipulation survey asking about their feelings during the interaction. Partners were then reunited and debriefed.

### 3.3. Measures

**Figure 3:**
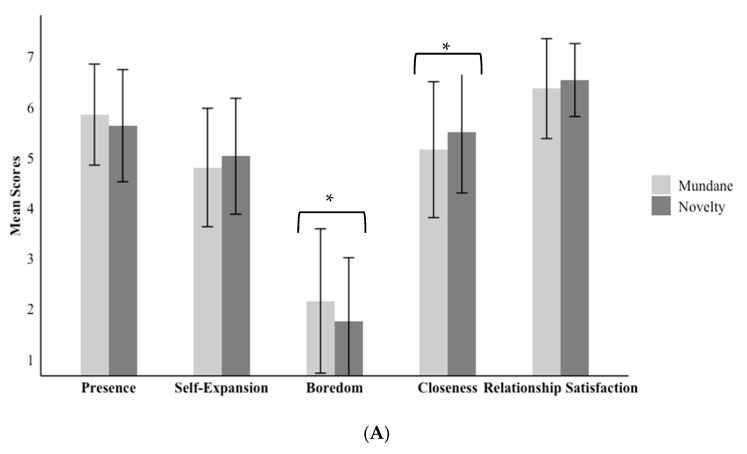

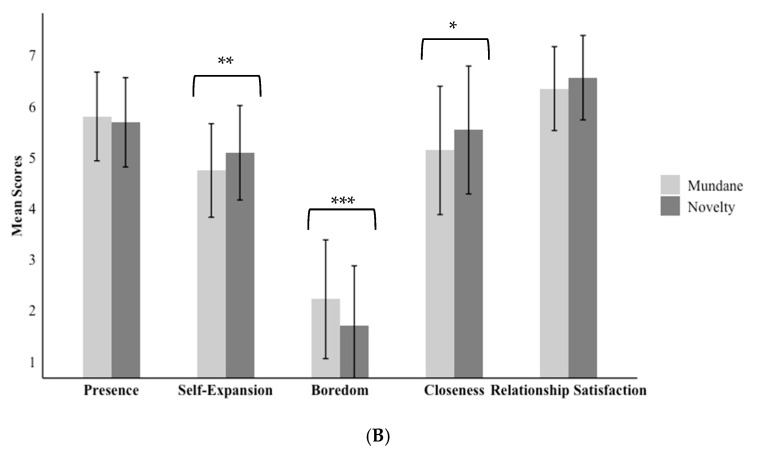
Descriptive statistics between conditions for Study 2 focal variables with (**A**) raw meansand (**B**) adjusted means (controlling for reports of immersion). Note. The error bars represent standard deviations, and significance is denoted with *** *p* < 0.001, ** *p* < 0.01, and * *p* < 0.05.

After exposure to the experimental condition, participants were asked to respond to a series of post-manipulation questions outlined below. Table 3 shows correlations between variables within each condition, and Figure 3 reports the means across conditions.

#### 3.3.1. Manipulation Check

Participants were asked two questions immediately after the interaction, which served as manipulation checks. The first questions read as follows: “You were asked to use virtual reality with your partner, what did you and your partner do?” with options including “Sat on a porch outside”, “Went on a virtual tour of the Swiss Alps”, or “Swam with sharks”. The second item asked participants to indicate, “How well do the following statements characterize the virtual experience you just had with your partner”?, “dull”, “exciting”, “fun”, and “interesting”, with response options ranging from 1 (*not at all*) to 7 (*extremely*). For the first item, participants who were in the mundane condition should have selected the first option, that they “sat on a porch outside”, and participants who were in the novelty condition should have selected the second option, indicating that they “went on a virtual tour of the Swiss Alps”. In line with our pre-registration, participants who incorrectly identified their condition were removed from data analyses. The second set of items (ratings of the experience as “dull”, “exciting”, “fun”, and “interesting”) served as additional manipulation checks (each item examined separately). We expected participants in the novelty condition to indicate that the experience was less dull and more fun, exciting, and interesting than participants in the mundane condition.

#### 3.3.2. Presence

Participants were asked to indicate presence using the same measure as in Study 1 (α novel = 0.78; α mundane = 0.77).

#### 3.3.3. Immersion

Post-interaction, participants were asked to answer two questions to assess their immersion during the interaction (e.g., “During our interaction, I was immersed in the environment”; “Our interaction was realistic”). Possible responses were on a 7-point scale (1 = *not at all*, 7 = *completely*), and the items were mean aggregated, with higher scores indicating greater perceived immersion (mundane *r* = 0.39, *p* < 0.001, novel *r* = 0.54, *p* < 0.001).

#### 3.3.4. Self-Expansion

Post-interaction, participants were asked to rate their perceptions of self-expansion with their partner during the interaction. Participants were provided the following instructions, “Thinking about the experience you and your partner just had together, answer each question according to the way you personally feel, using the following scale”. Participants were then asked to rate six items that were adapted from a measure of self-expansion (Lewandowski & Aron, 2002; e.g., “To what extent did this interaction feel like a new experience?”; “Did you learn new things during your experience?”; “How much did this experience expand your sense of the kind of person you are?”). Responses were provided on a 7-point scale (1 = *not very much*, 7 = *very much*), and the items were mean aggregated, with higher scores indicating higher self-expansion (α novel = 0.85; α mundane = 0.82).

#### 3.3.5. Boredom

Participants were asked to indicate how bored they were post-interaction using the scale employed in Study 1.

#### 3.3.6. Closeness

Participants were asked to indicate how close they felt to their partner after the interaction using the same item (i.e., the IOS) from Study 1 (Aron et al., 1992).

#### 3.3.7. Relationship Satisfaction

After the interaction, participants were asked to indicate their satisfaction with their partner using the same item as in Study 1.

#### 3.3.8. Experience with VR

Participants were asked to indicate their previous VR experience on a 4-point scale, as in Study 1. Many participants had some experience with VR, though, in Study 2, 36.6% of participants indicated no prior experience, 27.5% indicated using it once before, 30% indicated that they had used it a few times, and 5.6% indicated that they either owned a VR headset or used VR frequently (with one person, 0.3%, not answering the question).

### 3.4. Analytic Approach

As in Study 1, to examine whether people in the novel condition (compared to those in the mundane condition) reported greater self-expansion (H1), lower boredom, greater closeness, and relationship satisfaction (H2), we conducted a series of multilevel models with partners nested within couples. We tested whether the experimental condition (0 = mundane, 1 = novel) predicted our variables of interest. We then examined whether individuals in the novel VR condition reported less boredom and greater relationship quality post-interaction through self-expansion (H3). As in Study 1, to test our mediation models, we conducted a series of multilevel mediation models using the Monte Carlo Method for Assessing Mediation (MCMAM) (Selig & Preacher, 2024). We tested whether there were significant indirect effects, with indirect effects being deemed significant if the 95% CI did not contain zero in the analyses controlling for immersion. To account for individual differences in immersion, we controlled for the participant’s reports of immersion in the analyses. Importantly, reports of immersion did not differ by condition. That is, in an exploratory analysis, we found that reports of immersion did not differ in the novel (*M* = 5.22, *SD* = 1.24) and mundane (*M* = 5.42, *SD* = 1.10) conditions (*p* = 0.188). We conducted additional auxiliary exploratory analyses as in Study 1 to examine whether age, relationship length, or experience with VR moderated the effects of interest. The data and syntax needed to reproduce the results can be found on the OSF.

### 3.5. Results

#### 3.5.1. Manipulation Check

As expected, participants in the novelty condition reported a less dull (novel *M* = 1.70, *SD* = 1.26, mundane *M* = 2.04, *SD* = 1.45; *b* = −0.35, *SE* = 0.17, *t*(143.29) = −2.08, *p* = 0.039, CI [−0.68, −0.02]) and marginally more exciting experience than those in the mundane condition (novel *M* = 5.62, *SD* = 1.26, mundane *M* = 5.28, *SD* = 1.47; *b* = 0.35, *SE* = 0.18, *t*(142.93) = 1.90, *p* = 0.059, CI [−0.01, 0.70]). However, participants in the novel and mundane conditions did not differ in their reports of how fun (*b* = 0.05, *SE* = 0.16, *t*(143.03) = 0.32, *p* = 0.747, CI [−0.27, 0.37]) or interesting the experience was (*b* = −0.06, *SE* = 0.13, *t*(143.36) = −0.49, *p* = 0.626, CI [−0.32, 0.19]). Overall, participants found both conditions exciting, fun, interesting, and not dull, and when differences did emerge, they were small or marginal.

#### 3.5.2. The Effect of Novel Versus Mundane Virtual Experiences

As expected, participants in the novel (*M* = 5.64, *SD* = 1.11) and mundane conditions (*M* = 5.86, *SD* = 1.00) did not differ in their reports of perceived presence with their partner (*b* = −0.22, *SE* = 0.14, *t*(143.31) = −1.61, *p* = 0.109, 95% CI [−0.49, 0.05], given that both interactions were in VR. We then tested whether people in the novel condition reported more self-expansion than people in the mundane condition. Although participants in the novel condition did report higher self-expansion (*M* = 5.03, *SD* = 1.15) than those in the mundane condition (*M* = 4.81, *SD* = 1.17), differences between the conditions were not significant (*b* = 0.23, *SE* = 0.15, *t*(143.24) = 1.48, *p* = 0.140). However, consistent with our pre-registered predictions, people in the novel condition (*M* = 1.76, *SD* = 2.16) reported less boredom compared to those in the mundane condition (*M* = 2.16, *SD* = 1.43; *b* = −0.40, *SE* = 0.18, *t*(143.27) = −2.25, *p* = 0.026). Also, in line with our predictions, people in the novel condition (*M* = 5.51, *SD* = 1.21) reported greater closeness with their partner post-interaction compared to those in the mundane condition (*M* = 5.16, *SD* = 1.35; *b* = 0.35, *SE* = 0.17, *t*(144.01) = 2.03, *p* = 0.044). However, contrary to our predictions, there were no significant differences between groups on relationship satisfaction (*b* = 0.17, *SE* = 0.12, *t*(142.4) = 1.37, *p* = 0.17). Given that the condition did not significantly predict differences in self-expansion, we did not test indirect effects.

#### 3.5.3. Considering the Role of Immersion

In a series of exploratory analyses, we assessed the predicted associations controlling for participants’ reports of immersion during their virtual experience. Controlling for immersion, participants in the novel (*M* = 5.69 *SE* = 0.07) and mundane conditions (*M* = 5.80, *SE* = 0.07) did not differ in their reports of perceived presence with their partner (*b* = −0.12, *SE* = 0.11, *t*(143.69) = −1.06, *p* = 0.290, 95% CI [−0.33, 0.10]), as expected. However, effects did emerge for self-expansion, with participants in the novel virtual condition (*M* = 5.09, *SE* = 0.08) reporting more self-expansion after the interaction than people in the mundane condition (*M* = 4.74, *SE* = 0.08; *b* = 0.35, *SE* = 0.12, *t*(143.58) = 2.94, *p* = 0.004, 95% CI [0.11, 0.58]). In addition, controlling for immersion, people in the novel condition (*M* = 1.71, *SE* = 0.10) reported less boredom than people in the mundane condition (*M* = 2.22, *SE* = 0.10; *b* = −0.51, *SE* = 0.14, *t*(141.78) = −3.55, *p* < 0.001, 95% CI [−0.80, −0.23]). Participants in the novel condition (*M* = 5.54, *SE* = 0.10) also reported more closeness than those in the mundane condition (*M* = 5.14, *SE* = 0.11; *b* = 0.39, *SE* = 0.17, *t*(143.67) = 2.33, *p* = 0.021, 95% CI [0.06, 0.73]). Finally, although participants in the novel condition reported greater relationship satisfaction (*M* = 6.56, *SE* = 0.07) compared to those in the mundane condition (*M* = 6.35, *SE* = 0.07), the effect was not significant (*b* = 0.21, *SE* = 0.12, *t*(143.12) = 1.80, *p* = 0.075, 95% CI [−0.02, 0.44]), with both groups reporting high levels of relationship satisfaction (see Figure 3).

**Figure 4:**
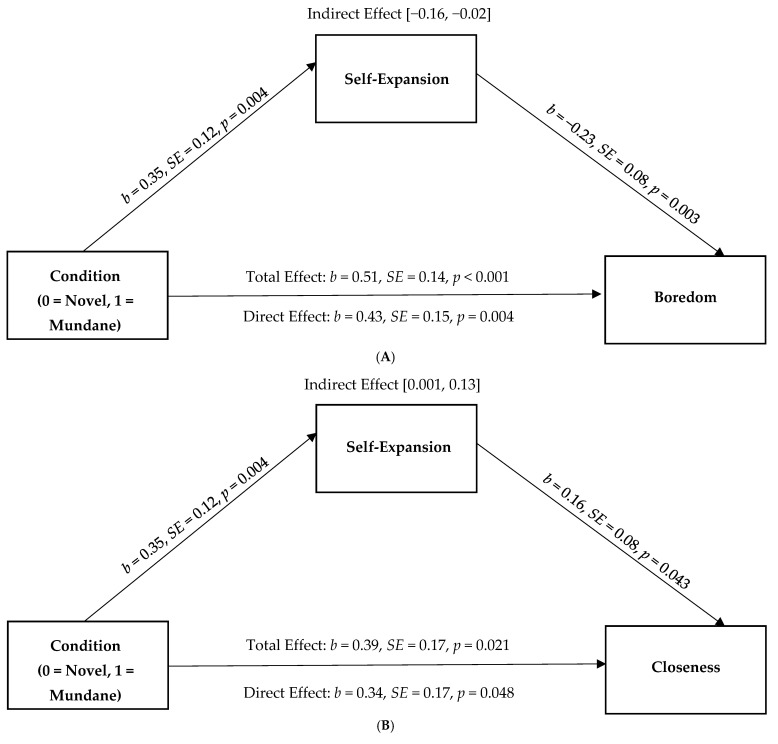

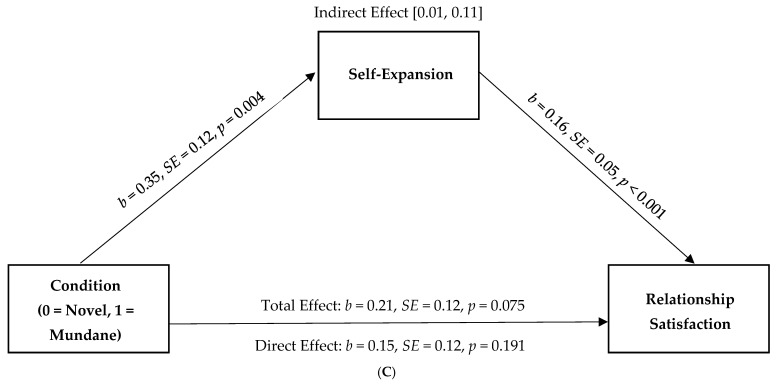
Self-expansion as a mediator of the association between the condition (0 = novel, 1 = mundane) on reports of (**A**) boredom, (**B**) closeness, and (**C**) relationship satisfaction controlling for immersion.

Lastly, we examined the mediation effects controlling for participant’s reports of immersion in the analyses. Results suggest that there were significant indirect effects of the condition on boredom (95% CI [−0.16, −0.02]), closeness (95% CI [0.001, 0.13]), and relationship satisfaction (95% CI [0.01, 0.11]) post-interaction through self-expansion (see Figure 4A–C). Although the effects are small, and the association between the condition and relationship satisfaction was not significant, this pattern of results suggests that novel (vs. mundane) experiences in VR can influence reports of self-expansion and have downstream effects on people’s reports of boredom, closeness, and satisfaction with their partners when accounting for immersion.

#### 3.5.4. Considering the Role of Relationship Length, Age, and Experience with VR

We conducted auxiliary analyses to test whether people in the novel (compared to the mundane) virtual experience differed on key demographics (e.g., age, relationship length) or experience with VR. We found no significant differences between conditions (in all cases, *p* > 0.05). Despite this, and consistent with Study 1, we explored if any of the effects differed based on participants’ age, length of the relationship, or past VR experience via moderation analyses. No significant interactions for relationship length, age, or experience with VR emerged (all *p* > 0.05).

### 3.6. Study 2 Discussion

Contrary to our hypotheses, differences did not emerge across conditions for self-expansion or relationship satisfaction. However, people in the novel VR condition reported lower boredom and greater closeness post-interaction compared to those in mundane VR condition. In exploratory analyses accounting for immersion (i.e., the extent to which they were engaged in the virtual environment), people in the novel condition reported greater self-expansion than those in the mundane condition and, in turn, lower boredom and greater relationship quality post-interaction. There were no significant differences based on relationship length, age, or previous experience with VR. The findings suggest challenges of manipulating virtual self-expanding experiences, as VR experiences (even more mundane ones) may be novel for most people, and hint that future research designing manipulations using VR should consider the role of immersion.

## 4. General Discussion

Self-expansion, which can be facilitated through shared novel activities in romantic relationships, is associated with lower boredom and greater relationship quality among romantic partners (Aron & Aron, 1996, 1997; Aron et al., 2022). However, in the globalized, modern world, many couples will experience periods in which they are apart (e.g., pursuing educational and vocational opportunities), and shared novel activities might be limited, thus hindering opportunities for couples to stay connected. Two dyadic experimental studies examined whether VR could simulate shared virtual couple activities. In Study 1, participants who engaged in a brief, novel virtual interaction with their partner (compared to participants who engaged in a brief, novel video interaction) experienced greater presence (i.e., felt they had a shared experience with their partner) and reported less boredom. Participants in the novel VR condition did not differ in their reports of self-expansion, relationship satisfaction, or closeness from those in the video condition. However, these effects worked indirectly through a greater sense of presence with a partner in the VR (versus) condition. In Study 2, couples engaged in either a novel and exciting or mundane experience in VR. Results were mixed. Contrary to our predictions, there were no differences between the condition on self-expansion or relationship satisfaction. However, participants in the novel VR condition reported less boredom and greater closeness post-interaction than those in the virtual mundane condition. In auxiliary analyses, we tested the role of immersion—the extent to which people feel engaged in the virtual environment and can block out the physical world. Past research demonstrates that there are individual differences in the extent to which people can block out the physical world and engage with a virtual world and this can be important for people’s experiences in VR (Berkman & Akan, 2019; Cummings & Bailenson, 2016; Maymon et al., 2023). Accounting for immersion in the virtual world, people in the novel VR condition reported greater self-expansion, less boredom, and greater closeness than those in the mundane VR condition. Additionally, holding immersion constant, results suggest that people who had a novel virtual interaction with their partner reported greater self-expansion and greater self-expansion was, in turn, associated with less boredom, greater closeness, and higher relationship satisfaction (although there were no main effects on relationship satisfaction). In Study 1, results suggested that people who were older and had less experience using VR benefitted more from novel interactions in VR than those who were younger or had more VR experience. There were no differences in Study 2 based on age or VR experience and no differences across studies for relationship duration.

### Examining Whether VR Can Enhance Relational Self-Expansion

Extending previous research examining self-expansion in relationships, the results from the current research demonstrate that shared novel and exciting activities in VR can provide opportunities for reducing boredom in romantic relationships. These findings are consistent with research showing that couples who engage in novel and exciting experiences together report less boredom (Aron et al., 2022). Contrary to our predictions and past research on self-expansion theory, the effects of novel virtual experiences on reports of self-expansion and relationship quality compared to novel video experiences (Study 1) or mundane virtual experiences (Study 2) were not significant in most instances (e.g., direct effects did not emerge, with the exception to Study 2, when immersion was controlled for). Thus, despite designing studies premised on self-expansion theory, the findings suggest that novel virtual experiences may help reduce boredom, although results for self-expansion and relationship quality are less promising.

The current research extends past research on self-expansion in relationships by examining novel experiences in VR. Most of the past research has focused on shared, in-person, novel and exciting activities among couples. Here, we tested whether self-expansion can be experienced virtually and have downstream consequences for relationship quality. The findings were mixed. The findings from Study 1 suggests VR has the potential (compared to video interactions) to simulate presence between partners, but there were no differences in self-expansion. Therefore, VR might provide new opportunities for partners to connect (i.e., feel like they are together) when in different geographical locations or when separated for extended periods of time. Also, given the range of activities available in VR (the full range was not tested in the current study), the findings from Study 2 suggest that some shared virtual activities (i.e., novel experiences such as a virtual gondola ride over the Swiss Alps) can foster closeness and reduce boredom compared to other virtual interaction (i.e., familiar scenery such as sitting on a virtual porch). However, more work is needed to understand how these experiences can promote self-expansion and further boost relationship quality.

The current research also has implications for researchers looking for novel ways to manipulate shared couples’ experiences in the lab. Past research on shared self-expansion in relationships has often focused on assessing naturalistic self-expanding experiences in couples’ daily lives (Coulter & Malouff, 2013; Harasymchuk et al., 2021; Muise et al., 2019) or manipulating novel (versus mundane) activities in the lab (Aron et al., 2000), the motivation to self-expand (Dys-Steenbergen et al., 2016), or need for self-expansion (Tsapelas et al., 2020). However, although more work is needed, VR presents an opportunity to develop experimental manipulations of shared activities that can occur in the lab but feel more realistic. Indeed, this research provides some initial evidence that VR technology can simulate the presence of a partner, unlike video chat, which leads to several possibilities for simulating or manipulating shared interactions or emotional experiences.

While the findings from these studies provide initial support for the promise of virtual reality for simulating a romantic partner’s presence (based on Study 1) and reducing boredom (based on Studies 1–2), more work is needed. More specifically, many of the effects across studies were small, and in some instances, indirect effects emerged in the absence of main effects. For instance, in Study 1, the main effect of the condition was only significant for reports of presence and boredom, not for reports of self-expansion, closeness, or relationship satisfaction post-interaction. Furthermore, there was no main effect on relationship satisfaction across both studies, despite past research showing a strong association between self-expansion and relationship satisfaction (Aron et al., 2022). The most consistent, strongest effect across studies was that the novel VR condition was associated with less boredom than the novel video condition (Study 1) and the mundane VR condition (Study 2). Although the effects of the condition on relationship quality did not emerge, it is possible that it was difficult to enhance relationship satisfaction because, in the current studies, couples were already highly satisfied; they did not have much room to grow. Future research might target more diverse samples and aim to refine the manipulations of shared experiences in VR.

Overall, these initial studies taught us some helpful lessons about using VR to manipulate self-expansion or other relationship constructs. From the results of Study 1, VR is able to simulate presence more than video interactions with a partner, which is important and suggests the ability to simulate shared couple activities or interactions in VR. Past research has shown that people report more presence (like they are really in the environment) in VR compared to other commonly used technologies (Baym et al., 2004; Froese et al., 2014; Meuleman & Rudrauf, 2018), but to our knowledge, the current research is the first to demonstrate participants’ perceptions of the presence of a romantic partner in VR.

It is also noteworthy that past research (Berkman & Akan, 2019; Howard et al., 2018; Maymon et al., 2023) has consistently demonstrated individual differences in people’s immersion in VR experiences, which could be important to account for in future research. More specifically, in Study 2, we found that many of the results of interest were non-significant when examined independently, though when we controlled for immersion, differences emerged across the groups, as discussed above. VR is widely considered to be more immersive than other technologies, allowing users to feel deeply “present” within a virtual environment, which in turn can lead to a stronger ability to induce emotions, both positive and negative, compared to traditional screens or other mediums (Li et al., 2022; Meuleman & Rudrauf, 2018; Riva et al., 2007). However, people differ in how much they engage with virtual environments and suspend their attention to the physical world (Berkman & Akan, 2019). Therefore, unique to VR studies, interactions should be designed with presence and immersion in mind, and these constructs should be assessed in VR research examining relational processes in VR.

## 5. Limitations and Future Direction

Across two experimental lab studies examining novel and exciting virtual interactions among romantic couples, it seems possible to enhance people’s perceptions of their partner’s presence by having them engage in novel virtual experiences (as seen in Study 1). Additionally, the finding from Study 2 suggests that people who engaged in a novel virtual experience with their partner did not report greater self-expansion unless immersion was controlled. However, when immersion was controlled for, people reported greater self-expansion and, in turn, reported lower boredom and higher levels of closeness and satisfaction compared to people who had a mundane virtual experience with their partner (Study 2). Importantly, in both studies, reports of boredom and relationship quality were assessed after one brief interaction in the lab, and we did not compare virtual experiences to in-person novel experiences. Future work could consider how couples might use VR outside of a lab environment and compare experiences in VR to in-person experiences. As such, using more ecologically valid methods (i.e., having geographically distant couples communicate with VR versus other technologies) could provide more insight into the potential uses of VR for connection with close others.

It is also important to note that the current studies included relatively homogenous samples of mostly mixed-sex, college-aged couples. Although the sample was racially diverse, participants were relatively young, and many were in the early stages of their relationship (e.g., on average, less than three years together). Past research shows that although opportunities for self-expansion are heightened in the early stages of relationships (Aron et al., 2022), the association between self-expansion and relationship quality can be stronger for couples in longer relationships (Muise et al., 2019). In the current studies, exploratory analyses indicate that the effects for reports of closeness differed based on age (e.g., an association between condition and closeness emerged among older but not younger participants in Study 1 only). Differences did not arise based on relationship length, although there was insufficient diversity in relationship length (as well as a restricted age range). We also could not adequately test whether the association between virtual self-expansion and reports of boredom and relationship quality were widely generalizable (e.g., across diverse sexual and relationship orientations). Thus, these findings should only be generalized to a heterosexual, college-aged sample of couples in relatively short-term relationships. Future research would benefit from examining the effects among a more diverse and representative sample and across a wider age and relationship length range, as some of the auxiliary analyses suggested that differences may emerge among those who are older or who have been with their partner for more time.

Finally, a practical consideration of this work is finding appropriate stimuli to manipulate shared novel activities. We used an existing application (i.e., Alcove) and piloted different experiences (mostly related to travel) from the options. At the time that this research was being conducted (2022–2023), Alcove was one of the main options for multi-player VR applications that would allow people to have a shared experience with an interaction partner while restricting access to others. In future work, researchers could program their own stimuli to control the interaction’s specific features. For example, in a recent study of individuals, the researchers designed a manipulation to induce fear in which participants walked along a plank in the lab while in a VR headset. In the fear group, participants were 80 stories high. In contrast, in the control group, they were at ground level (El Basbasse et al., 2023). This is an excellent example of how VR can be used to conduct ecologically valid experiments and successfully induce emotions. Moving forward, there are many exciting directions to pursue, but related to the current study and thinking about dyadic research in VR, the early research examining the misattribution of arousal (e.g., when people mistakenly attribute their physiological arousal to an interaction partner on a scary bridge versus a safe bridge) could be replicated using virtual reality with a similar design to the fear manipulation.

## 6. Concluding Remarks

In the globalized, modern world, where couples spend less time with each other and are more likely to live apart than in past generations, finding ways for couples to stay connected is more important than ever. Based on the findings from Study 1, engaging in novel, virtual interactions with a romantic partner (compared to video interaction) simulated greater partner presence, which, in turn, was associated with higher reports of self-expansion, less boredom, and higher relationship quality (e.g., relationship satisfaction and closeness) post-interaction. Furthermore, across the two experimental studies, we found that engaging in a novel virtual interaction was consistently associated with lower reports of boredom, and was, at times, associated with greater closeness (e.g., Study 2). However, contrary to our predictions, engaging in novel virtual experiences with a partner was not associated with reports of self-expansion or relationship satisfaction in either study. For example, in Study 2, in which we compared mundane to novel virtual interactions, we found no association between condition and self-expansion, though when we controlled for reports of immersion, this effect became significant, and self-expansion was, in turn, associated with lower boredom and greater relationship quality. According to this research, while virtual novel experiences with a romantic partner may help partners feel like they are in each other’s presence and protect against boredom, the condition has limited effects on self-expansion and relationship quality. This suggests that research examining the effects of VR on relationships should carefully consider the role of presence and immersion in the study design and the effects. Overall, this research supports the idea that VR might offer people new opportunities to engage in shared activities with close others and raises some promising directions for future research using VR to manipulate shared experiences. That said, more work is needed to understand when and under what conditions virtual experiences can promote self-expansion and bolster the relationship quality among romantic partners.

## Figures and Tables

**Table 1:** Demographic information for Study 1 and Study 2.

	Study 1	Study 2
	Mean (SD) or N (%)	Mean (SD) or N (%)
Age	20.20 (3.81)	20.30 (3.64)
Relationship Length	2.22 (2.50)	1.83 (1.77)
Gender		
Man	92 (49.46%)	128 (44.60%)
Woman	93 (50.00%)	156 (54.36%)
Non-Binary	-	3 (1.05%)
No response	1 (0.50%)	-
Race/Ethnicity		
White	69 (37.10%)	70 (24.39%)
South Asian	29 (15.59%)	74 (25.78%)
East Asian	-	30 (10.45%)
Black	31 (16.7%)	30 (10.45%)
Latin American	16 (8.60%)	23 (8.01%)
Native American/First Nation	1 (0.54%)	-
Bi- or multi-racial	19 (10.22%)	30 (10.45%)
Self-identified	21 (11.29%)	43 (14.98%)
Sexual Orientation		
Heterosexual	139 (74.73%)	216 (75.26%)
Lesbian/Gay	4 (2.15%)	5 (1.74%)
Bisexual	32 (17.20%)	48 (16.72%)
Pansexual	6 (3.23%)	4 (1.39%)
Queer	1 (0.54%)	1 (0.35%)
Questioning	2 (1.08%)	8 (2.79%)
Asexual	-	1 (0.35%)
No response	2 (1.08%)	2 (0.70%)
Relationship Status		
Casually dating	4 (2.15%)	34 (11.85%)
Seriously dating	164 (88.17%)	227 (79.09%)
Engaged	4 (2.15%)	-
Common-law	5 (2.69%)	6 (2.09%)
Married	8 (4.30%)	17 (5.92%)
No response	1 (0.54)	3 (1.05%)
Past VR Experience		
None	36 (19.4%)	105 (36.6%)
Used it once	51 (27.4%)	79 (27.5%)
Used it a few times	73 (39.2%)	86 (30.0%)
I own a VR/use it regularly	15 (8.1%)	16 (5.6%)
Missing	11 (5.9%)	1 (0.3%)

**Table 2:** Correlations among focal variables in Study 1.

	1	2	3	4	5	6
Novel Video Condition						
1. Presence	-					
2. Self-Expansion	0.45 ***	-				
3. Boredom	−0.35 **	−0.28 **	-			
4. Closeness	0.52 ***	0.34 ***	−0.42 ***	-		
5. Relationship Sat.	0.40 ***	0.38 ***	−0.32 ***	0.46 ***	-	
6. Relationship Length	−0.05	−0.12	0.15	−0.09	−0.08	-
Novel VR Condition						
1. Presence	-					
2. Self-Expansion	0.48 ***	-				
3. Boredom	−0.41 **	−0.36 **	-			
4. Closeness	0.60 ***	0.51 ***	0.45 ***	-		
5. Relationship Sat.	0.43 ***	0.44 ***	−0.30 ***	0.57 ***	-	
6. Relationship Length	−0.02	−0.15	0.22 *	−0.11	−0.14	-

Note: *** *p* < 0.001, ** *p* < 0.01, * *p* < 0.05. Rel Sat = relationship satisfaction.

**Table 3:** Correlations among focal variables in Study 2.

	1	2	3	4	5	6
Mundane VR Condition						
1. Immersion	-					
2. Self-Expansion	0.52 ***	-				
3. Boredom	−0.51 ***	−0.34 ***	-			
4. Closeness	0.23 **	0.27 ***	−0.24 **	-		
5. Relationship Sat.	0.40 ***	0.34 ***	−0.43 ***	0.38 ***	-	
6. Relationship Length	−0.20 *	−0.16	0.26 **	0.02	−0.12	-
Novel VR Condition						
1. Immersion	-					
2. Self-Expansion	0.69 ***	-				
3. Boredom	−0.51 ***	−0.50 ***	-			
4. Closeness	0.19 *	0.18 *	−0.22 **	-		
5. Relationship Sat.	0.18 *	0.26 **	−0.26 **	0.32 ***	-	
6. Relationship Length	0.03	0.05	−0.05	0.02	0.07	-

Note: *** *p* < 0.001, ** *p* < 0.01, * *p* < 0.05. Rel Sat = relationship satisfaction.

## Data Availability

Study 1 and Study 2 were pre-registered on the Open Science Framework (OSF). We have also provided our anonymized data and syntax on the OSF. As this paper is under review, the data component is currently private, though we are happy to grant access to it upon request. We are committed to making all these components public with the final published paper to increase transparency and reliability and facilitate the replicability of the current findings.

## Note

1 In the pre-registration for Studies 1–2, we indicated that regression analyses would be used for the analyses. However, as the data comprise romantic couples, we modified the approach to use mlms instead, as this analytic strategy accounts for the interdependence among partners. Results for regression analyses overlap substantially with the mlm analyses.
